# A multidisciplinary approach to the diagnosis of antisynthetase syndrome

**DOI:** 10.3389/fmed.2022.959653

**Published:** 2022-09-14

**Authors:** Matthew Wells, Sughra Alawi, Kyaing Yi Mon Thin, Harsha Gunawardena, Adrian R. Brown, Anthony Edey, John D. Pauling, Shaney L. Barratt, Huzaifa I. Adamali

**Affiliations:** ^1^Department of Rheumatology, North Bristol NHS Trust, Bristol, United Kingdom; ^2^Bristol Interstitial Lung Disease Service, North Bristol NHS Trust, Bristol, United Kingdom; ^3^Immunology Laboratory, North Bristol NHS Trust, Bristol, United Kingdom

**Keywords:** antisynthetase syndrome, myositis, interstitial lung disease, arthritis, connective tissue disease, multidisciplinary team

## Abstract

Antisynthetase syndrome is a subtype of idiopathic inflammatory myopathy, strongly associated with the presence of interstitial lung disease. Diagnosis is made by identifying myositis-specific antibodies directed against aminoacyl tRNA synthetase, and relevant clinical and radiologic features. Given the multisystem nature of the disease, diagnosis requires the careful synthesis of subtle clinical and radiological features with the interpretation of specialized autoimmune serological testing. This is provided in a multidisciplinary environment with input from rheumatologists, respiratory physicians, and radiologists. Differentiation from other idiopathic interstitial lung diseases is key; treatment and prognosis differ between patients with antisynthetase syndrome and idiopathic interstitial lung disease. In this review article, we look at the role of the multidisciplinary team and its individual members in the initial diagnosis of the antisynthetase syndrome, including the role of physicians, radiologists, and the wider team.

## Introduction

Antisynthetase syndrome (ASyS) is a subtype of idiopathic inflammatory myopathy (IIM), strongly associated with the presence of interstitial lung disease (ILD) and aminoacyl tRNA synthetase antibodies (antisynthetase antibodies). Although the presentation is heterogeneous, the classical clinical features include but are not limited to the triad of ILD, myositis, and arthritis (1, 2). The antisynthetase antibody target antigen, aminoacyl tRNA synthetase, resides within the cytoplasm of cells and plays a role in the translation of mRNA into protein, yet the antibody's role in the aetiopathogenesis is undetermined (3).

Given prominent pulmonary and musculocutaneous clinical features, ASyS represents a true interdisciplinary multisystem disease presenting to both respiratory and rheumatology services. The diagnosis requires the careful evaluation of clinical features, radiology, and expertise in the interpretation of serological tests. This is best provided in a multidisciplinary setting wherein accurate diagnosis and treatment decisions are shared among clinical and diagnostic teams. This review will evaluate the multidisciplinary approach to the diagnosis of ASyS, referring to the clinical and serological features observed in the spectrum of disease.

## Classification and epidemiology

Classification criteria are developed to identify a homogeneous cohort of patients from an existing population group of interest for recruitment into research studies. Two sets of classification criteria for ASyS were published in 2010 and 2011 based on expert consensus, both acknowledging the presence of aminoacyl tRNA synthetase antibodies as necessary for classification alongside the presence of specific clinical features (4, 5). To date, eight antibodies have been described in the literature: anti-Jo1, the most frequently observed in up to 88% of ASyS patients (6), anti-PL7, anti-PL12, anti-OJ, anti-EJ, anti-KS, anti-Zo, and anti-SC (3) (Table 1). The 2017 European League Against Rheumatism/American College of Rheumatology (EULAR/ACR) classification criteria for adult and juvenile idiopathic inflammatory myopathies and their major subgroups do not acknowledge ASyS as a distinct entity, and anti-Jo1 antibody was the only myositis-specific antibody included in the final classification criteria due to the underrepresentation of other antibodies (7). It has been shown that the inclusion of the other antisynthetase antibodies in the EULAR/ACR classification criteria improves sensitivity for ASyS (8), but there are concerns that this may compromise specificity (9, 10).

**Table 1:** Myositis-specific and myositis-associated autoantibodies; the antisynthetase syndrome associated antibodies are in bold with each specific target aminoacyl tRNA synthetase enzyme in brackets.

**Myositis-Specific autoantibodies**	**Myositis-Associated autoantibodies**
**Antisynthetase:anti-Jo1** (histidyl); **anti-PL7** (threonyl); **anti-PL12** (alanyl); **anti-OJ** (isoleucyl); **anti-EJ** (glycyl); **anti-Ks** (asparaginyl); **anti-Zo** (phenylalanyl); **anti-SC** (lysyl)	PM/Scl 75/100
MDA5	U1 RNP
Mi2	U1/U2 RNP
SRP	U3 RNP
SAE-1	Ro52
NXP2	Ku
TIF1γ	

MDA5, melanoma differentiation-associated protein 5; Mi2, Nuclosome remodeling-deacetylase; NXP2, Nuclear matrix protein 2; PM/Scl, Polymyositis/Scleroderma; SRP, signal recognition particle; SAE-1, SUMO-activating enxyme subunit 1; TIF1γ, Transcription intermediary factor 1 gamma; URNP, Uridine rich ribonucleoprotein.

Presently, no data driven or validated classification criteria exist for ASyS and definitions vary widely across the literature and are poorly evidenced (11). Project CLASS is an ongoing large multicentre international effort funded by EULAR and ACR aiming to develop a validated criteria set for ASyS. This large-scale study should lead to more representative ASyS patient recruitment in future research studies and may further our understanding of the influence of the antisynthetase antibody serology on disease phenotype.

The epidemiology of ASyS is unclear. A study in inner-city Manchester revealed a mean incidence of IIM (as defined by the European League Against Rheumatism/American College of Rheumatology EULAR/ACR classification criteria) of 17.6/1,000,000 person-years, of which 28% were identified as ASyS by expert consensus (12). Females are affected more frequently than males, and the mean age at onset ranges from 43 to 60 years (13). Data suggest that black patients may have more frequently occurring and severe ILD but ASyS epidemiology does not otherwise appear to be influenced by ethnicity (14, 15). Older studies with varying disease definitions, failure to include the more recently discovered antisynthetase antibodies, and lack of the aforementioned data-driven classification criteria have impacted reported epidemiology.

## Clinical features

The classical triad of ILD, myositis, and arthritis was reported at first presentation (defined as the development of all three features within 3 months of first symptom onset) in only 5% of the large international American and European Network of Antisynthetase Syndrome (AENEAS) cohort, including anti-Jo1, anti-PL7, anti-PL12, anti-EJ, and anti-OJ antibody-positive patients and 15% of a Chinese cohort of antisynthetase antibody-positive ILD patients (16, 17). In the AENEAS study, the onset was predominantly a single triad feature in all included antibody subgroups, suggesting the condition can frequently present as isolated ILD, myopathy, or arthritis. It concluded that presentation and disease course between antibody groups is broadly similar. However, this observation is inconsistent across the literature; a 2014 meta-analysis including 3,487 patients suggested a higher prevalence of ILD in non-anti-Jo1 and myositis in anti-Jo1 patients (18).

A significant proportion of patients in the AENEAS cohort developed additional clinical features during the follow-up (16). Beyond the classical triad, there are several other commonly occurring features as outlined below, alongside other less well-reported examples of organ involvement that can significantly impact morbidity and mortality.

## Pulmonary disease

ILD has a prevalence of 67 to 100% in ASyS, with cough and dyspnoea the most common symptoms (19, 20). Some reports suggest a greater prevalence of ILD in anti-PL7 and anti-PL12 positive patients when compared to the more frequently encountered anti-Jo1. The John Hopkins myositis center cohort observed isolated lung involvement at disease onset in anti-PL7 and anti-PL12 patients at 56 and 65%, respectively, compared to only 26% among patients with anti-Jo1 disease (14). After 3.4 years of follow-up, 19% of anti-PL7 and 30% of anti-PL12 patients had failed to develop myositis. Hervier's cohort described more severe ILD in anti-PL7 and anti-PL12 diseases with lower forced vital capacity (FVC) and concomitant poorer survival when compared to anti-Jo1 cases (21).

This contrasts with the findings of the previously mentioned AENEAS cohort (16). Further analysis of the anti-Jo1 positive patients in AENEAS showed that 21% presented with isolated pulmonary symptoms. At follow-up (median 72 months), ILD was observed in 82% of anti-Jo1 patients, 20.8% of whom were asymptomatic. Other cohorts have also failed to show differences in ILD severity according to serology (22).

The timing of presentation of ILD in the disease course can be variable. Pulmonary symptoms may present early, as seen in “lung dominant disease,” develop simultaneously alongside other symptoms, or may appear later in the course of the disease (16, 23). We have seen locally that patients referred to respiratory services with ILD are more likely to have amyopathic disease concurring with the findings of Hervier and colleagues (21, 24); interestingly, our respiratory cohort did not have significantly different lung function abnormalities compared to those presenting to rheumatology.

Other forms of ILD must be considered. Idiopathic pulmonary fibrosis (IPF) patient demographics frequently differ from those of ASyS and other connective tissue disease-associated ILD (CTD-ILD), being predominantly male and of older age at onset (25). However, a significant proportion of ASyS and IPF patients may overlap demographically, and this can contribute to missed or delayed diagnosis (24).

Pleural involvement has been reported in ASyS; a recent study including 93 patients observed that 42.2% had pleural effusions (26). Anti-Jo1 patients appeared less likely to suffer pleural effusions than non-anti-Jo1 patients. Serositis is not widely reported in the literature but given its prevalence in this cohort, this is an area that needs further evaluation.

## Assessment of lung disease

### Physiology

We would recommend spirometry, lung volumes, diffusion capacity, and a 6-min walk test in all patients with suspected ILD. These measurements of lung physiology help assess the severity and pattern of respiratory impairment.

Physiological impairment in ILD-related ASyS is a restrictive lung pattern with a decreased diffusion capacity for carbon monoxide (DLco). Serial PFTs are helpful for disease monitoring, prognostication, and response to treatment (27, 28); a relative decline of FVC of 10% or greater, or 5 to 10% with clinical deterioration has been considered to suggest ILD progression (29).

A disproportionate reduction in the FVC compared to the radiological lung interstitial abnormalities may indicate diaphragmatic or respiratory muscle weakness; hence, measurement of maximal inspiratory pressure (MIP) and maximal expiratory pressure (MEP) may be helpful. MIP represents the strength of the inspiratory muscles including the diaphragm, and MEP reflects the strength of the expiratory muscles. Normal MIP usually excludes significant diaphragmatic involvement (30).

Performance status can be assessed and quantified using the 6-min walk test, with saturation probe measurement to identify dips in oxygen saturation. Performance can be influenced by non-pulmonary aspects of ASyS such as myopathy and Raynaud's and should be interpreted accordingly.

### Imaging

High-resolution computed tomography (HRCT) plays a critical confirmatory role in the diagnosis of ILD. Ground glass opacities (hyper-attenuated areas with preserved bronchial and vascular markings), linear opacities, reticulations, and traction bronchiectasis (bronchial distortion caused by mechanical traction by the fibrosis) are frequently seen in ASyS. Areas of consolidation (hyper-attenuated areas with loss of normal bronchial and vascular markings) also may be seen (3, 31, 32).

**Figure 1:**
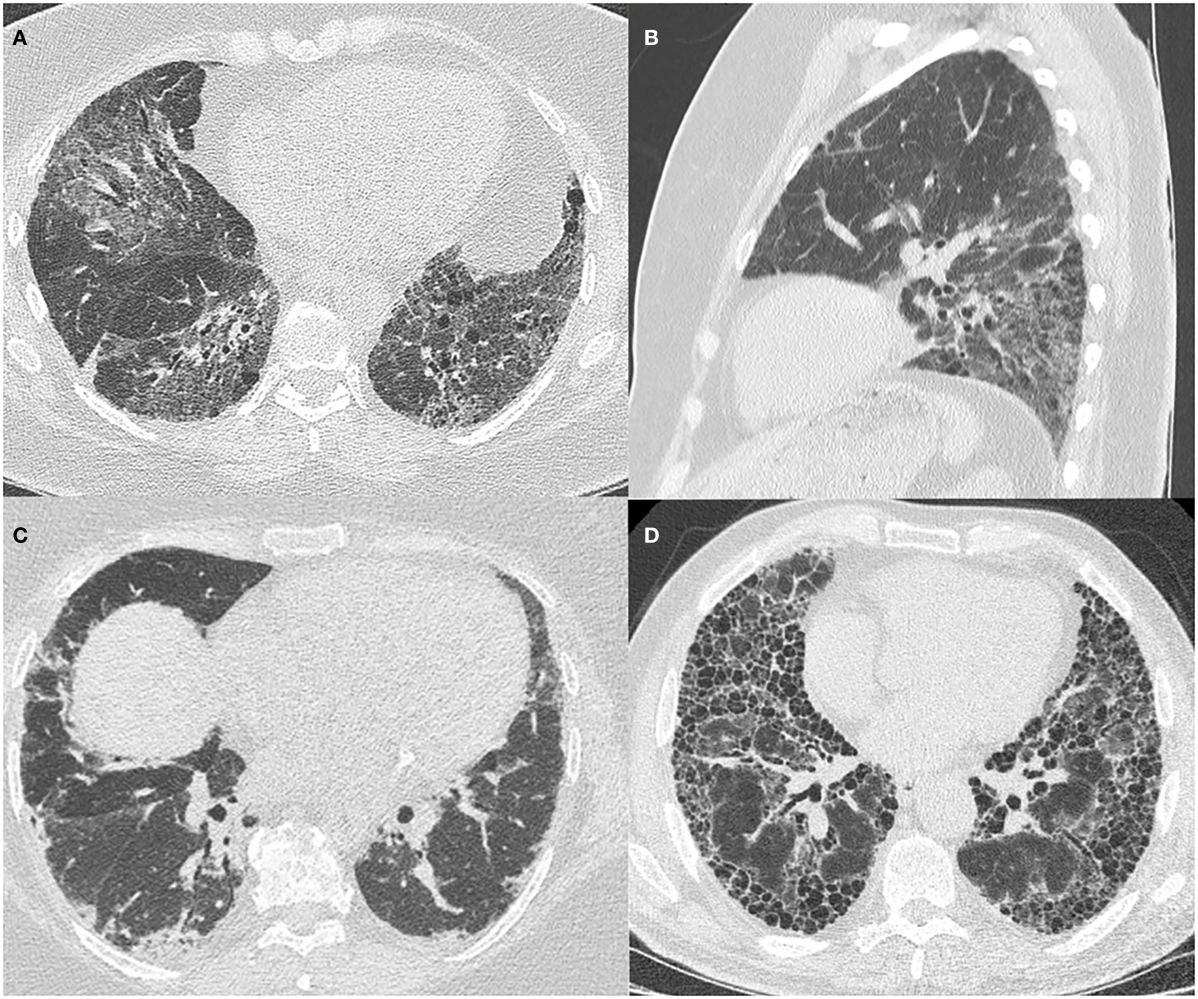
HRCT images of ILD patterns seen in ASyS. All patients are positive for anti-Jo1 antibody and have an MDT diagnosis of ASyS. **(A)** Axial view of fibrotic NSIP demonstrating patchy ground glass opacification with reticulation and traction airway dilatation; **(B)** Sagittal view of fibrotic NSIP demonstrating the lower zone predominance in the same patient; **(C)** Axial view of organizing pneumonitis with patchy, peripheral basal predominant consolidation; **(D)** Axial view showing UIP pattern with subpleural basal predominant reticulation and extensive honeycombing evident.

Common radiological patterns described in ASyS are non-specific interstitial pneumonitis (NSIP), organizing pneumonitis (OP), and NSIP/OP overlap (Figure 1). Less commonly, usual interstitial pneumonitis (UIP) and acute interstitial pneumonitis (AIP) have been reported (3, 24, 32–34). There is no clear association between the different HRCT patterns and the different antisynthetase antibodies, and all patterns are seen across all antibody subtypes.

However, multiple cohort studies have shown a broad spectrum of ILD manifestations on HRCT scans. In a large Chinese cohort, the spectrum varied from an OP pattern predominantly seen in anti-Jo1 positive ASyS with an NSIP pattern evident in anti-PL12 and anti-PL7. In anti-EJ patients, an OP pattern was most predominantly seen (35). Other smaller cohorts have shown overlap NSIP/OP patterns to be more prevalent in anti-Jo1 and anti-PL12 (36).

### Other tests

ILD guidelines recommend routine serological testing in all patients with newly identified ILD, including for anti-nuclear antibodies (ANA) by immunofluorescence, with onward referral to rheumatology, if there are extra-pulmonary connective tissue disease (CTD) features or positive serologies (25). Often no invasive investigations are required and the presence of ILD with circulating antisynthetase antibodies is sufficient for multidisciplinary diagnosis.

Bronchoalveolar lavage (BAL) is unlikely to change a clinical and serological diagnosis of CTD-ILD (37). While BAL fluid cytology has been described in various subtypes of ILD, findings are non-specific, and therefore the role of BAL in ASyS assessment is limited. It may be performed in cases of non-UIP type ILD presenting without CTD features or if infection or pulmonary hemorrhage are considered in the differential diagnosis (38).

Lung biopsies including open, video-assisted thoracoscopic surgery and cryobiopsy, are not routinely performed. The main histological patterns identified are NSIP and OP. UIP and diffuse alveolar damage (DAD) are less frequently seen; one study has suggested that UIP is more common in anti-PL12 disease (39). Histopathological findings usually correlate with the HRCT pattern (40). Multidisciplinary consensus with improved imaging technology has significantly reduced the need for invasive procedures (41). When there is a failure to reach a consensus diagnosis, lung biopsy may remain an option to support the overall multidisciplinary impression.

## Myopathy

Myopathic muscular weakness was observed in 91% of ASyS patients in the cross-sectional EuroMyositis registry (20). The AENEAS cohort describes myositis present in 56.1% of anti-Jo1 patients at disease onset, of which 15.2% were subclinical, detected by elevation of muscle enzymes or EMG alone without clinically apparent weakness. This rose to 82.1% at 72 months follow-up, with the only significant difference between antibody subgroups being more infrequent myositis in anti-PL12, with 30% at onset rising to 43% at 37.5 months follow-up (16). More frequent myositis in anti-Jo1 has also been observed in other cohorts (21). Amyopathic patients are more likely to present to respiratory services, where co-existent myalgia or elevated creatine kinase (CK) was relatively uncommon at 30% in Barratt et al.'s cohort (24).

The spectrum of myopathy may range from subclinical disease to significant proximal weakness, causing difficulties rising from a seated position, climbing staircases, or reaching overhead cupboards (42). Persistent myalgia and muscular tenderness are reported in 30.4 to 88.9% of cases (2, 43, 44). Weakness may be elicited on examination and should be quantified using manual muscle testing of eight muscle groups (MMT8 score); this is a standardized power assessment tool for myositis patients used by rheumatologists with excellent reproducibility (45).

### Assessment of myopathy

Investigations should aim to confirm the clinical impression of myopathy, with simultaneous scrupulous assessment for the ancillary features of ASyS described below, which can represent 20 to 25% of all IIM cases (46). This initially consists of the measurement of serum levels of the muscle enzymes creatine kinase (CK) and aldolase. Cardiac Troponin T (cTnT), lactate dehydrogenase (LDH), and alanine transferase (ALT) can also be elevated during skeletal muscle inflammation. Levels of CK elevation vary with a mean level of 4,288 U/L, but as high as 22,820 U/L in one Japanese cohort of ASyS patients presenting with limb muscle weakness (47), yet these muscle enzymes are neither sensitive nor specific and it should be cautioned that this Japanese study recruited patients with clinically apparent myopathy, thus excluding clinically amyopathic ASyS patients. Muscle enzymes may be normal, and while they can be useful markers of disease activity, they do not necessarily correlate with myositis activity, especially in more chronic diseases. British Society of Rheumatology guidelines recommend testing for myositis antibodies in patients with suspected inflammatory myopathy (48), and in the correct clinical context, these may be diagnostic.

**Figure 2:**
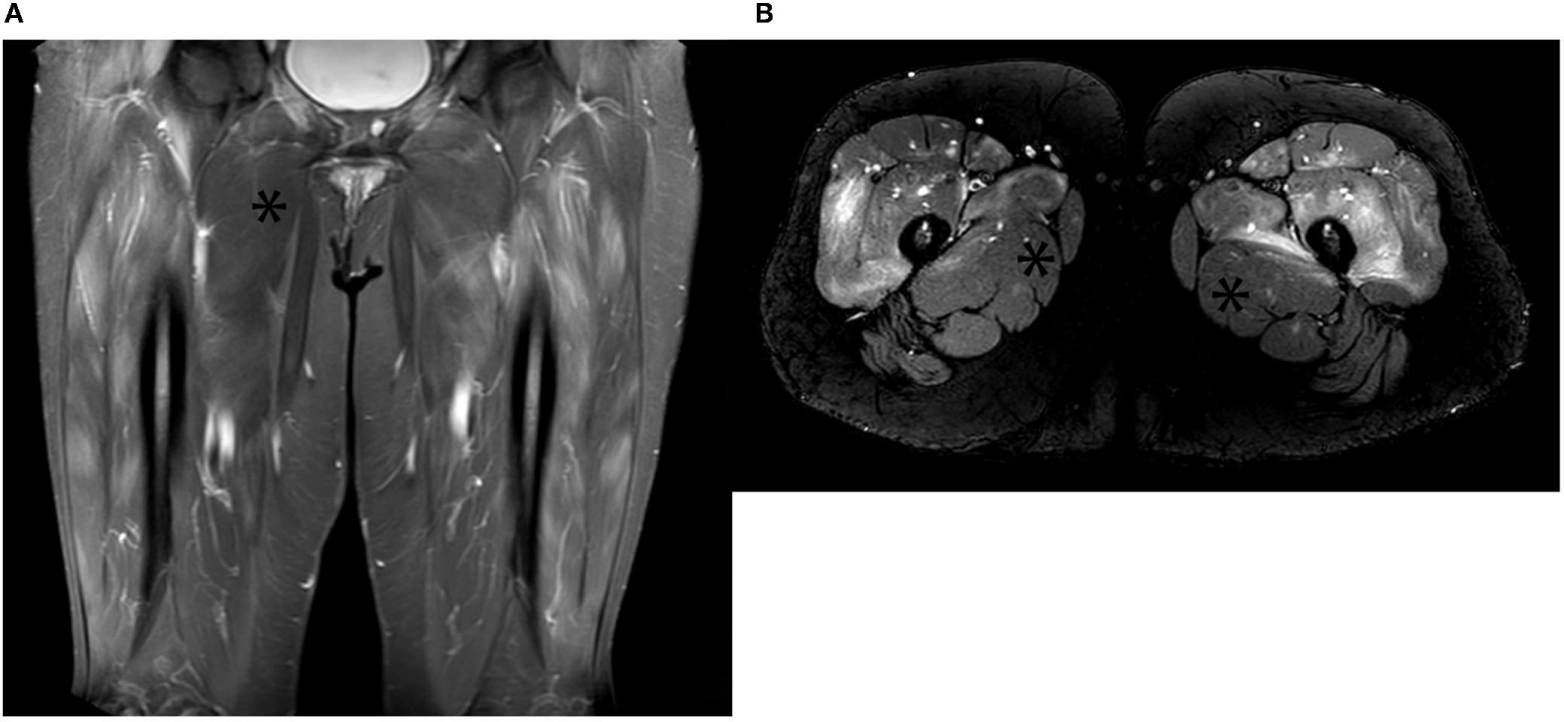
**(A)** Contrast-enhanced coronal STIR images of bilateral upper thigh muscles (anterior compartment) with extensive hyperintensities indicating features of muscle edema. The asterisk demonstrates the spared adductor muscle; **(B)** Axial STIR images of the same patient showing classical edema signal pattern (high signal intensity) in the anterior compartment of both upper thigh muscles. Inflamed muscles demonstrate contrast enhancement. The asterisk demonstrates the spared adductor muscle.

If there is clinical uncertainty, multidisciplinary support from neurophysiologists and musculoskeletal radiologists can facilitate confirmation of myopathy in IIM. Electromyography shows early recruitment of motor unit potentials with spontaneous activity, a highly suggestive pattern for IIM (49). Sensitivity is reported to be as high as 93.8% for ASyS, but this deteriorates with concomitant glucocorticoid exposure, and findings lack specificity for the IIM subtype (50). Magnetic resonance imaging (MRI) with T2 weighted imaging (T2WI) and short tau inversion recovery (STIR) sequencing detecting muscular and fascial edema and fatty replacement can be seen in as many as 65% of ASyS patients (51) (Figure 2). More chronic myositis changes are seen with T1 weighted imaging (T1WI) as atrophy with fat replacement (52).

Muscle biopsy is rarely required to confirm a diagnosis of ASyS. It can, however, be indispensable in ruling out alternative causes of myopathy, such as muscular dystrophies, metabolic, infectious, toxin/drug-mediated disease, and inform the decision to commence immunosuppressive treatment regimes (53). MRI can be used to target and increase muscle biopsy sensitivity (52).

When performed, muscle biopsy with findings of mononuclear cell infiltrates and muscle fiber necrosis are supportive of a clinical impression of IIM and may help determine dermatomyositis (DM)/inclusion body myositis (IBM) and Immune-mediated necrotizing myopathy (IMNM) (53, 54). Necrotizing myopathy is the most common finding in antisynthetase antibody-positive patients. When DM and IBM samples were excluded, myofiber HLA DR expression is highly specific in muscle biopsy specimens from antisynthetase antibody-positive patients, although the utility of this finding is yet to be fully understood (55). With the growing availability of commercial serological assays for identifying antisynthetase antibodies, there is less need for muscle biopsy for the diagnosis of ASyS. Muscle histology still has an important role in cases where the clinical picture of IIM is incomplete, for example, if there are no obvious CTD features or where a circulating myositis-specific antibody cannot be identified (48).

## Arthritis

Arthritis is widely documented as a symmetrical, non-erosive polyarthritis of the small joints of the hands and feet in ASyS (56), mimicking seronegative inflammatory, rheumatoid, and CTD-associated arthritides. It is a non-specific symptom occurring in 18 to 55% of IIM patients and can be the initial presenting feature in 24 to 66% of ASyS patients; this contributes to diagnostic delay as patients can be initially diagnosed and managed as inflammatory arthritis (57–59).

Analysis of the AENEAS cohort suggests that patients who develop arthritis during the course of the disease are more likely to suffer from accompanying CTD features such as mechanics hands, Raynaud's phenomenon, and fever (see sections). In contrast, patients presenting with early-onset arthritis have more of a rheumatoid-type presentation (60). When followed-up, hand X-ray lesions were seen to develop in 41.9% of ASyS patients irrespective of serology (61). These lesions included periarticular calcifications, erosions, and subluxations. In patients with inflammatory joint disease and ILD, ASyS should be considered alongside the more common rheumatoid lung disease (57, 62).

## Raynaud's phenomenon

**Figure 3:**
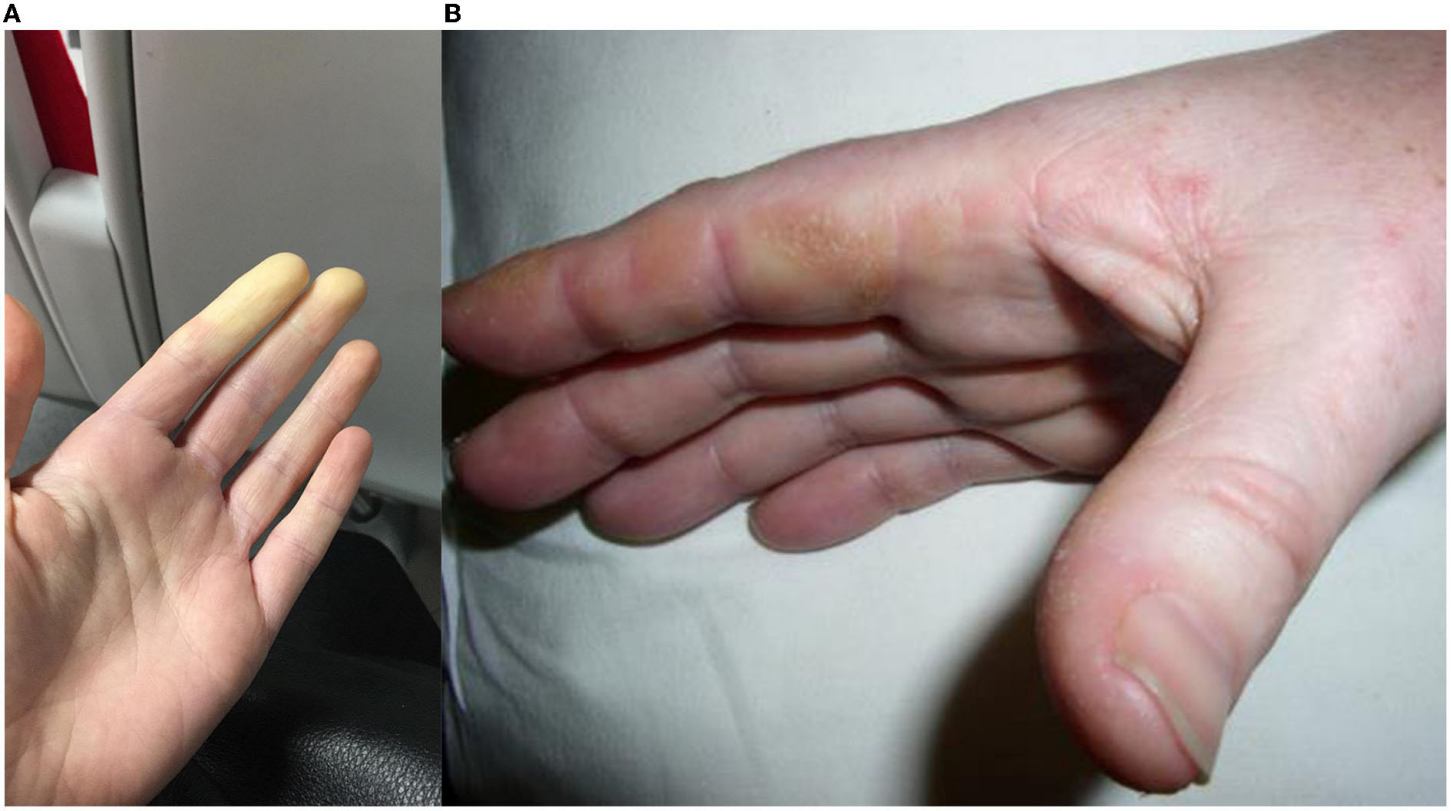
**(A)** Raynaud's phenomenon, initial vasoconstriction causing pallor, which usually progresses to cyanosis then reactive hyperemia in triphasic Raynaud's; **(B)** Mechanic's hands, hyperkeratotic, fissured skin predominantly visible over the radial border of the index finger.

Raynaud's phenomenon is typically a cold-provoked peripheral ischemia manifested clinically by a digital color change from white (Figure 3A) to blue and then red (classical “triphasic” Raynaud's), and can be primary or secondary to another disease process. Prevalence is self-reported as 4.6% among adults in Great Britain, most of whom are primary (63). Primary Raynaud's occurs without a disease association, typically with onset in young (under 30 years old) females with symmetrical episodic attacks precipitated by the cold without ulceration or tissue compromise, and often with negative or non-specific ANA tests (64).

Age over 30 years at onset may raise suspicion for secondary Raynaud's phenomenon, alongside more frequent attacks, more significant pain during onset, and asymmetry than with primary Raynaud's. It is seen associated with CTD, such as systemic lupus erythematosus (SLE), mixed-connective tissue disease (M-CTD), and systemic sclerosis (SSc), as well as ASyS, where it was reported in 51% of the 512 cases in the EuroMyositis registry (20). While severe secondary Raynaud's may progress to digital ischemia and ulceration in SLE and SSc, this is seldom seen in primary Raynaud's and ASyS (65).

At a microvascular level, nail-fold videocapillaroscopy (NVC) is abnormal in ASyS compared to a control population of primary Raynaud's patients (66). In this study, ASyS patients demonstrated reduced capillary density, more severe microhemorrhages, and more giant capillaries than the control population. These findings are not specific to ASyS and may be seen in other CTD, including SSc. Further research needs to be performed to clarify any potential role for NVC, it may play a role in the diagnosis and monitoring of ASyS in future (67).

## Mechanics hands and DM-like rash

Mechanics hands refer to roughening and cracking of the skin of the lateral aspects of the fingers, typically the radial border of the index and ulnar border of the thumb (Figure 3B). This was reported in 38% of ASyS cases included in the EuroMyositis registry (20). Mechanics hands do not appear to have any prognostic value and are also seen in DM and OM, albeit less frequently (68). On biopsy, an interface psoriasiform dermatitis is seen (69).

A DM-like rash is observed in 32 to 44% of patients with antisynthetase antibodies (18). This includes psoriasiform-type lesions extending across the dorsum of the hand morphologically similar to those observed in mechanics hands, Gottron papules, and heliotropic rash.

While these rashes may be part of the heterogeneous ASyS disease spectrum, they may also be suggestive of DM or OM and MSA/MAA may help to determine the serological diagnosis. Such differentiation between ASyS and DM is clinically and prognostically important given differing profiles of malignancy risk and evidence suggesting that ILD associated with ASyS may carry a better prognosis than when associated with other IIM (70).

## Fever

Fever when present should prompt investigation for infection, although the reported prevalence in ASyS ranges from 21 to 66% (23, 71). A Chinese cohort study identified more pyrexial episodes with acute phase response in anti-PL7 (50%) and anti-Jo1 (30%) positive cases, with a more inflammatory phenotype predictive of rapidly progressive ILD (RP-ILD) (72). The large 2014 meta-analysis demonstrated more fever in non-anti-Jo1 patients as opposed to anti-Jo1 positive patients (18). Neither the AENEAS nor EuroMyositis cohorts present data on fever prevalence.

## Other CTD manifestations

Several other manifestations of the disease are described in the literature. Cardiac involvement, including pulmonary hypertension (PH), was seen in 7.9% of a multicenter French retrospective study, with subsequent significant impact on survival, although felt to be secondary to co-existent ILD (73). The authors conclude that clinicians should consider routine screening for PH in ASyS patients. This should be suspected when the degree of dyspnoea is disproportionate to the burden of disease on HRCT or if there is a strong SSc phenotype. Antisynthetase antibodies are sometimes present in patients with SSc, highlighting the significant clinical overlap that may exist between IIM and SSc spectrum disorders (74). DLco may also be disproportionately low compared to other lung function testing parameters. PH is presumed secondary to ILD, however, subgroups of ASyS patients with PH appear to have severe PH with relatively mild ILD, raising suspicion for a pulmonary vasculopathy as seen in SSc patients with group 1 PAH (75).

Another study reported a prevalence of myocarditis of 3.4% without any link to antibody specificity but all cases presented with active myositis (76). As mentioned previously, cTnT may be elevated in active myositis, whereas cardiac Troponin I (cTnI) is more specific for myocardial disease and although not universally available, it may be a helpful non-invasive screening test for primary cardiac involvement (77).

Analysis of a small cohort looking at the manifestations of disease not included in the classification criteria suggested that dysphagia (27%), DM rash (24.3%), and sicca symptoms (24.3%) commonly occur (78).

## Autoimmune serology

The presence of ANA is seen in association with various “ANA-associated rheumatic diseases” including the IIM alongside SLE, SSc, Sjogren's syndrome (SS), and M-CTD. They are also seen in other non-rheumatic diseases (autoimmune liver disease) and are present in up to 13.3% of otherwise healthy individuals at a 1:80 dilution (79). ANA is a collective term for a heterogeneous group of antibodies directed against cell components, traditionally within the nucleus. However, many target antigens are located outside the nucleus leading some commentators to suggest that they may be better considered “anti-cell antibodies” (80).

Over recent decades, the discovery of multiple MSA and MAA within the ANA spectrum has revolutionized the understanding of IIM and allowed researchers to identify distinct serologically defined phenotypes. Detection of these specific autoantibodies now plays a vital role in the diagnosis of ASyS. Immunoprecipitation (IP) is the gold standard detection method for autoantibodies, given its superior sensitivity and specificity. However, it is only available in a few specialized centers, limiting its use to research rather than clinical settings (81, 82).

**Figure 4:**
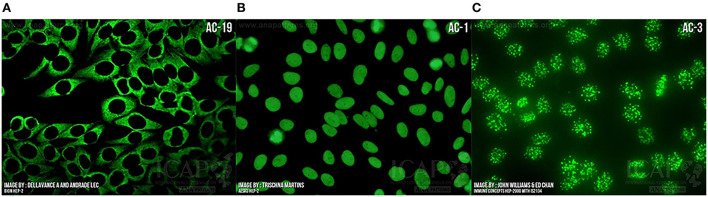
Indirect immunofluorescence demonstrating “anti-nuclear” antibody bound to **(A)** Cytoplasm as seen in ASyS; **(B)** Homogeneous nuclear binding as seen in SLE, autoimmune hepatitis, and juvenile idiopathic arthritis; **(C)** Centromere binding as seen in limited cutaneous SSc with anti-centromere antibody positivity. Images courtesy of ANApatterns.org—with permission.

Screening for the presence of ANA by immunofluorescence (IF) on HEp2 cell lines is recommended by the ACR as the gold standard ANA screening technique (83). HEp2 cells display a range of antigens not included in other ANA detection techniques and also present the advantage of providing the cellular location of any target antigens as well as titer levels (Figure 4). If ANA by IF is positive, more specific tests are performed on the patient serum to identify antigen specificity. Historically, this comprises assays for the six most common antibodies, which only include one antisynthetase antibody (anti-Jo1).

Importantly, screening by HEp2 ANA and other techniques may be negative but the presence of a cytoplasmic staining pattern under fluorescent microscopy suggests the presence of antibodies. This was well-demonstrated in a 2017 study showing that 82% of ANA-negative ASyS patients had positive cytoplasmic staining on IF and this pattern had high sensitivity and specificity for the presence of antisynthetase antibodies (84). In the setting of a high pre-test probability, extended assaying for disease-specific antibodies should be performed on these samples, such as the myositis blot. The results of these tests should then be interpreted in conjunction with the IF staining pattern and clinical correlation. Antisynthetase antibodies should correlate with a cytoplasmic staining pattern on IF and this improves the positive predictive value (85).

IF is labor intensive and dependent upon the availability of trained technicians who are able to report staining patterns. Despite efforts to standardize testing, reporting of patterns and differing cell lines in use can all limit IF (86). Subsequently, other techniques such as enzyme-linked immunosorbent assays (ELISA), chemiluminescence, and bead technology are also in use, with the advantages of reduced costs, higher throughput, and less dependence on trained laboratory staff. Given the significantly reduced antigen profile as compared to IF on HEp2 cells, sensitivity is lacking and no information on antibody binding patterns is provided. These differing antigen profiles and limitations must be considered when relying on non-IF detection methods and MSA should be tested for where there are cutaneous, pulmonary, or vascular (Raynaud's) features suggestive of IIM, even if ANA testing is negative by these techniques.

**Figure 5:**
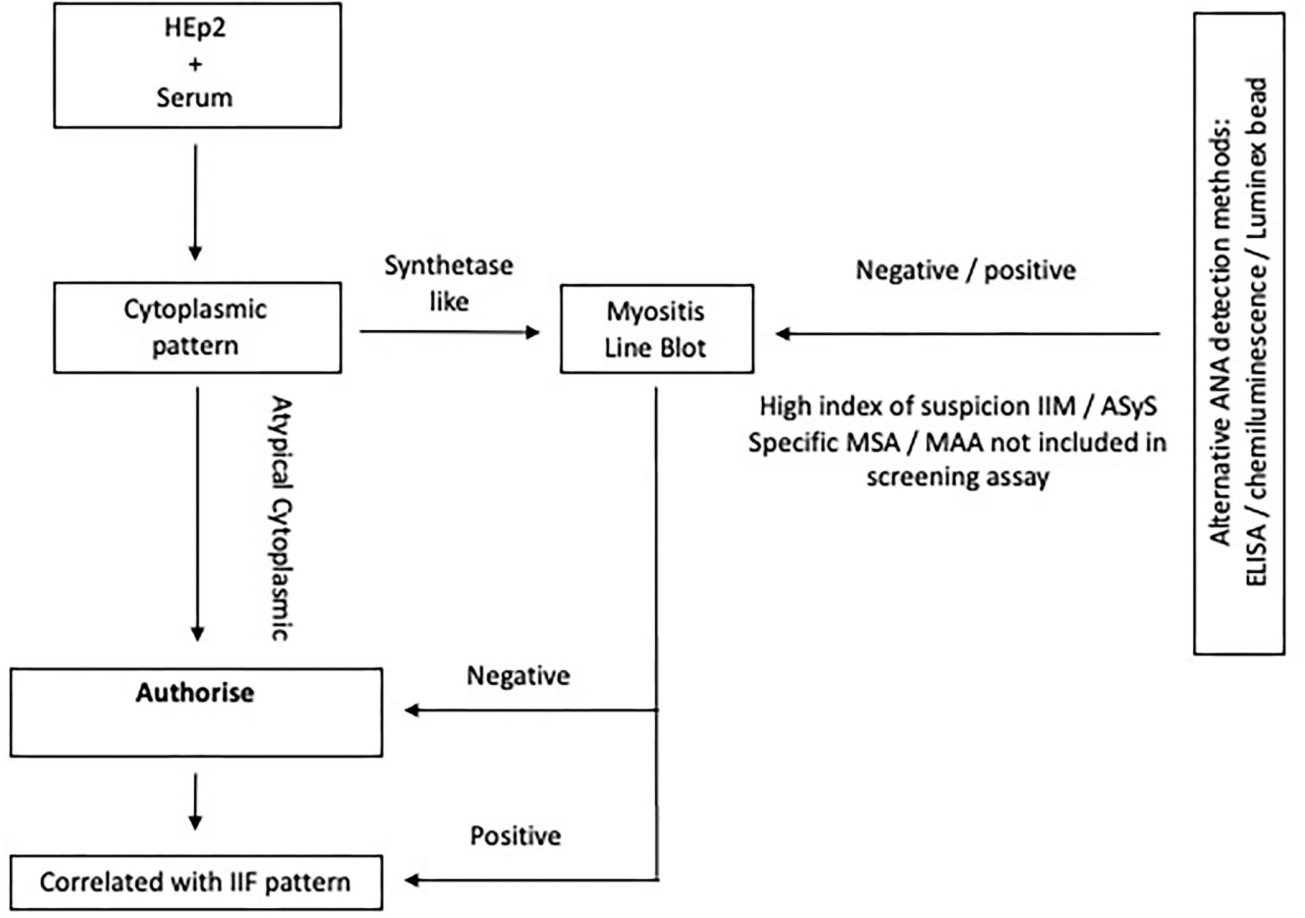
Algorithm depicting ANA assaying in ASyS. Extended testing is advised in the context of a cytoplasmic pattern on IF. If alternative ANA screening methods are used and suspicion of ASyS or IIM remains high, it may be appropriate to perform extended testing, especially when certain potentially relevant MSA or MAA are not included in the screening antigen profile and there are cutaneous, pulmonary, or vascular features of IIM.

Further extended testing for MSA and MAA, which include the antisynthetase antibodies, is performed by line or dot blot technology (Figure 5). Recent work suggests that these tests have poor sensitivity for the less common anti-OJ, anti-EJ, and anti-PL7 antibodies (87). Commercial immunoblots are also liable to false positives; the previous study reported that 16.1% of healthy controls tested positive for an MSA. Results must always be interpreted in the context of the immunofluorescence pattern, clinical-radiological presentation, and strength of positivity (88).

Notably, the anti-Ro52 antibody is found in a significant proportion of ASyS patients and appears to correlate with the presence and severity of ILD (89). It is detected in concert with the aminoacyl tRNA synthetase antibodies on an extended myositis blot and may be informative in terms of prognosis and guiding initial treatment strategies as well as a surrogate marker of ASyS (90).

## Multidisciplinary team assessment

The initial secondary care assessment of ASyS patients usually takes place in respiratory or rheumatology clinics given the wide prevalence of arthritis, myositis, and pulmonary disease. However, the condition may also initially present to neurology or dermatology, given the possibility of prominent weakness or cutaneous disease at the time of onset.

Increasingly, combined rheumatology and respiratory clinics are becoming more common place (91). These allow for expert assessment for clinical features suggestive of autoimmune rheumatic disease and interpretation of complex autoimmune serological tests by a rheumatologist. Respiratory specialist presence facilitates diagnosis with the aid of advanced physiological assessment and radiological investigations. The exclusion of idiopathic, drug-related, and environmental causes for lung disease is of paramount importance (92).

Clinical features should be assessed and confirmatory investigations arranged as above with findings carefully documented. Combined clinics not only influence clinical and therapeutic outcomes but are also a time and cost-effective way of providing quality care to ASyS patients (91).

If after this assessment, ASyS or alternate CTD-ILD is suspected, cases should be referred onwards for discussion in regular multidisciplinary team meetings. This gold standard multidisciplinary team (MDT) meeting provides an evidence-based diagnostic and management decision platform (25, 93–95). The diagnostic quorum includes members who have a special interest in CTD-ILD. The inclusion of rheumatologists reduces the need for invasive procedures to secure diagnosis (96). Thoracic radiologists play a key role in determining ILD patterns and expert interpretation of HRCT scanning. Radiology is essential; data show that clinicians change their diagnosis based on HRCT findings in up to 51% of ILD cases (97). Respiratory physicians with a specialist interest in ILD can clinically correlate radiological findings and pulmonary physiology.

**Figure 6:**
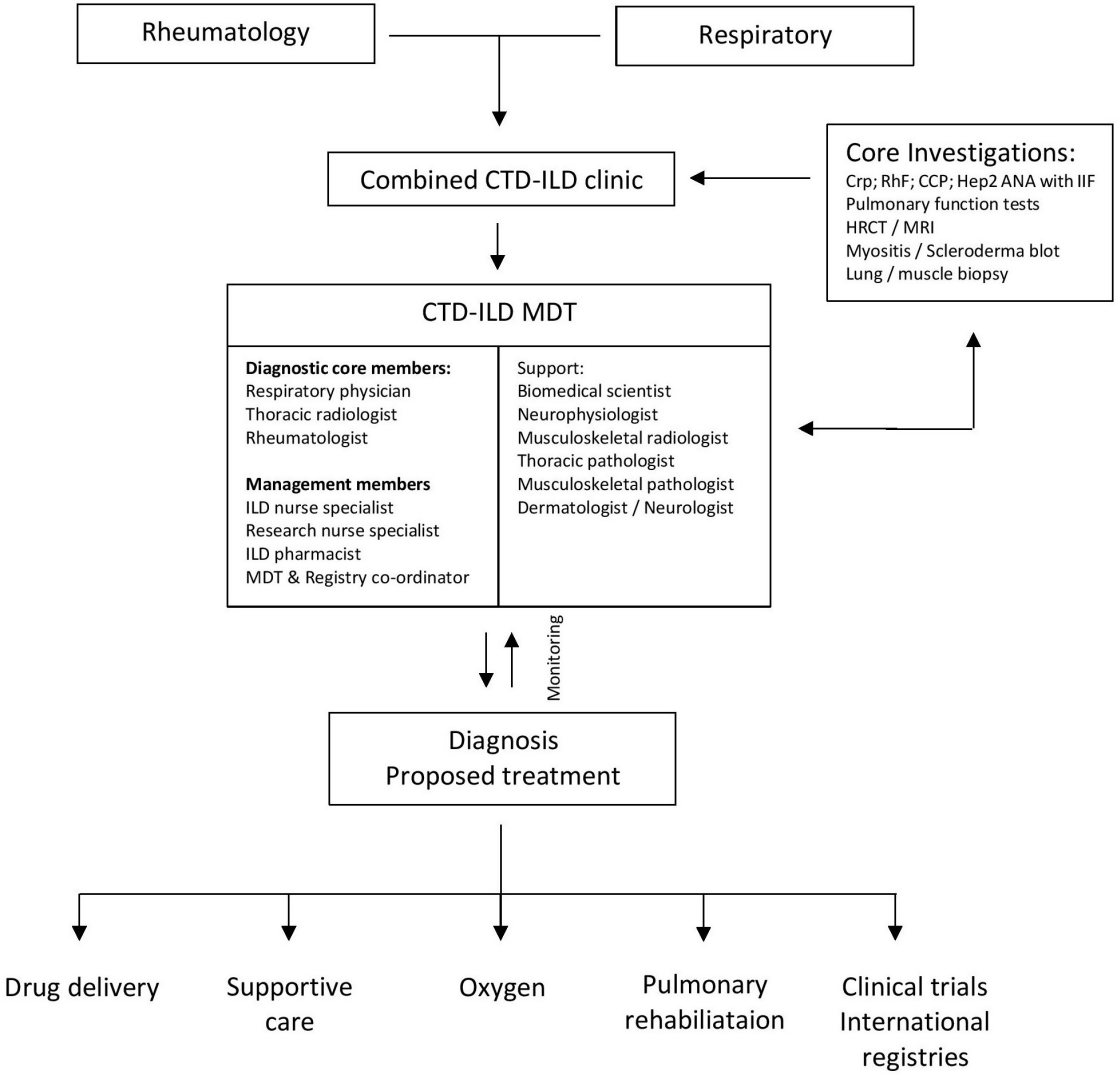
Multidisciplinary working within the CTD-ILD multidisciplinary team.

Our experience also supports the value of a formal CTD-ILD MDT comprising ILD nurse specialists, dedicated ILD pharmacists, research nurses, a registry coordinator, and auxiliary specialties who facilitate delivery of oxygen therapy, pulmonary rehabilitation, high-cost drugs, clinical trials, management of registries, symptom control, and palliation (Figure 6).

The centralization of these specialist services into a regional MDT facilitates center experience, networking, case identification, and biobanking. Using teleconferencing facilities, regional CTD-ILD MDT allows access for local district hospitals to secure multidisciplinary input. Electronic documentation of demographics, disease parameters, investigations, and drug management allows data to be presented and shared at this meeting. The ability of external referrers to be present and provide oral information and subtle nuances in these meetings impacts securing an accurate diagnosis. Diagnosis and recommendations can be made and shared electronically within the primary and secondary care system. This model of care reduces the impact on overstretched tertiary services and helps develop local expertise in the diagnosis and management of CTD-ILD patients (98). Local provision of patient care impacts long-distance traveling and inconveniencing patients who may be significantly ill.

In the correct clinical context, invasive procedures are rarely required (41). For incomplete cases, such as those without a detectable circulating antibody or atypical radiology, the MDT is well-placed to arrange surgical lung or muscle biopsies to better inform any decision to treat with immunosuppressive therapy. As discussed earlier, histology may be supportive but is non-specific for ASyS. It can, however, be crucial in ruling out alternative causes. In the presence of a compatible clinical phenotype, cytoplasmic staining pattern on IF, and detectable Ro52, it may be possible to make an MDT diagnosis of ASyS without invasive procedures or a specific detectable antisynthetase antibody. Consequently, it is important to recognize that real-world MDT diagnoses do not always correlate with current classification criteria and this limitation should be considered when reviewing patients.

ASyS patients should be followed-up regularly and any change in clinical condition should prompt rediscussion within the MDT, wherein decisions regarding disease progression, treatment response, and new organ involvement can be raised and discussed among all members. Importantly, clinical trial nurse specialists should advise on the potential eligibility of patients for existing available trials.

## Malignancy in antisynthetase syndrome

The presence of concomitant malignancy is well-reported in IIM, especially in patients with DM. A 2021 meta-analysis suggests that any ASyS-related autoantibody is significantly associated with a lower risk of cancer when compared to other IIM cases; however, few studies were included (99). The same meta-analysis concluded that older age (over 40 years), male sex, and the presence of dysphagia are factors that may increase malignancy risk that could be relevant to an ASyS cohort. The International Myositis Assessment and Clinical Studies (IMACS) group is currently developing guidelines for assessing cancer risk, screening, and follow-up in IIM.

## Conclusion

ASyS patients represent a clinically and serologically distinct subgroup of both IIM and ILD patients. Within the broad spectrum of ASyS, it is unclear to what degree serology influences presentation and prognosis. It may be that anti-Jo1 patients represent a more musculoskeletal phenotype of disease with less severe ILD, whereas non-anti-Jo1 patients have more aggressive ILD, possibly associated with decreased survival (18). This heterogeneity across cohorts is likely confounded by differing study methodologies, case definitions, antibody detection techniques and thresholds, and patient assessments with variable follow-up (1, 18, 43).

Diagnosis is a gestalt process—considering clinical features, imaging characteristics, and immunology, usually with aminoacyl tRNA synthetase antibody detection and appropriate cytoplasmic staining on IF. This should be confirmed within a diagnostic and management CTD-ILD MDT setting. If the clinical picture is incomplete or serological testing is inconclusive, the threshold for targeted muscle or lung biopsy should be lowered to ensure that important differential diagnoses are not missed and inappropriate immunosuppression is not offered.

The MDT has evolved to become a platform not only for the initial assessment but also with upcoming new treatments and the introduction of antifibrotic agents, an opportunity to hone down the diagnosis and provide holistic and complete care for ASyS patients.

## Author contributions

All authors listed have made a substantial, direct, and intellectual contribution to the work and approved it for publication.

## Funding

Funding for the publication of this article has been kindly provided by the Bristol Interstitial Lung Disease Research Fund.

## Conflict of interest

HG has received speaker and advisory fees from Boehringer-Ingelheim. JDP has received personal support from and undertaken consultancy work for Janssen, Astra Zeneca, Permeatus Inc, Boehringer-Ingelheim and Sojournix Pharma. SLB has undertaken consultancy work and received speaker honoraria from Boehringer-Ingelheim. The remaining authors declare that the research was conducted in the absence of any commercial or financial relationships that could be construed as a potential conflict of interest.

## Publisher's note

All claims expressed in this article are solely those of the authors and do not necessarily represent those of their affiliated organizations, or those of the publisher, the editors and the reviewers. Any product that may be evaluated in this article, or claim that may be made by its manufacturer, is not guaranteed or endorsed by the publisher.
